# Clinical Benefits of KRAS/GNAS Gene Mutation Analysis in Addition to Morphology and Conventional Cyst Fluid Testing in Differentiating Pancreatic Cysts

**DOI:** 10.3390/jcm14248671

**Published:** 2025-12-07

**Authors:** György Gyimesi, Bánk Keczer, Péter Rein, Miklós Horváth, Bálint Gellért, Tamás Marjai, Enikő Tóth, Ákos Szűcs, Attila Szijártó, Tamás Barbai, Eszter Székely, István Hritz

**Affiliations:** 1 School of Doctoral Studies, Semmelweis University, 1082 Budapest, Hungary; 2 Department of Gastroenterology, Spital Thurgau AG, 8596 Münsterlingen, Switzerland; 3 Department of Surgery, Transplantation and Gastroenterology, Semmelweis University, 1082 Budapest, Hungary; keczer.bank@semmelweis.hu (B.K.); rein.peter.laszlo@semmelweis.hu (P.R.); marjai.tamas@semmelweis.hu (T.M.); toth.eniko@semmelweis.hu (E.T.); szucs.akos@semmelweis.hu (Á.S.); szijarto.attila@semmelweis.hu (A.S.); 4 Department of Surgery, Transplantation and Gastroenterology, Division of Interventional Gastroenterology, Semmelweis University, 1082 Budapest, Hungary; horvath.miklos@semmelweis.hu (M.H.); gellert.balint@semmelweis.hu (B.G.); hritz.istvan@semmelweis.hu (I.H.); 5 Department of Pathology, Forensic and Insurance Medicine, Semmelweis University, 1082 Budapest, Hungary; barbai.tamas@semmelweis.hu (T.B.); szekely.eszter@semmelweis.hu (E.S.)

**Keywords:** pancreatic cystic neoplasm, intracystic CEA, intracystic glucose, KRAS, GNAS, mutation analysis

## Abstract

**Objectives:** Pancreatic cystic lesions (PCLs) are increasingly detected due to the widespread use of imaging techniques. The identification of pancreatic mucinous cysts is especially important since these carry a risk of malignant transformation and require follow-up or surgical resection. The aim of this study was to determine the diagnostic yield of the molecular analysis of K-RAS (Kirsten RAt Sarcoma virus) and GNAS (Guanine Nucleotide-binding protein, Alpha Stimulating protein activity) gene mutations in pancreatic cyst fluid (PCF) obtained by endoscopic ultrasound (EUS)-guided fine needle aspiration (FNA). **Methods:** In this prospective trial, we assessed the sensitivity, specificity, and positive and negative predictive values of K-RAS and GNAS mutation analysis in differentiating mucinous versus non-mucinous cysts and the subsequent impact on decision-making in daily clinical practice. The reference standard used comprised the combination of morphology on cross-sectional imaging and EUS, string sign, cyst fluid cytology, intracystic carcinoembryonic antigen (CEA), and glucose levels, with subsequent correlation of surgical pathology in resected cases. Fluid samples of 47 cysts obtained by EUS-FNA over a 39-month period were analyzed. Mutation analysis of KRAS (exon 2) was performed in all cases, and additionally, GNAS (exon 8) in 28 cases using Sanger sequencing. **Results:** 33 out of 47 PCLs were classified as mucinous cysts and 14 as non-mucinous cysts defined using conventional standards, including morphological characteristics, string-sign, cytology, cyst fluid testing, and histology in resected cases. Of these 33 mucinous cysts, KRAS mutation was detected in 14 samples. A further 23 mucinous lesions were additionally tested for GNAS mutation, which was detected in 10 of the 23 cysts. A 42.4% sensitivity for KRAS and 43.5% for GNAS mutation analysis was calculated, with a specificity of 92.9% and 100%, respectively, for detecting mucinous lesions. The clinical management was altered through the genetic testing results in one single case. **Conclusions:** In this cohort, K-RAS and GNAS mutational analysis in cyst fluid did not improve the detection of mucinous pancreatic cysts significantly after conventional testing. However, the method may be useful due to its high specificity in uncertain cases.

## 1. Introduction

Pancreatic cystic lesions are increasingly detected due to the frequent use of imaging technologies. The incidence of PCLs ranges from 2.6% to 13.5% in asymptomatic patients, with an increasing prevalence seen with older age [1,2,3]. PCLs represent a wide spectrum of entities from benign to malignant lesions. Non-neoplastic cysts (pseudocysts) and benign neoplastic cysts, such as serous cystadenomas (SCAs), display no malignant potential and therefore require only clinical follow-up. Alternatively, premalignant lesions such as mucinous cystic neoplasms (MCNs) and intraductal papillary mucinous neoplasms (IPMNs) have the potential to progress to invasive adenocarcinoma, and, therefore, surveillance of these lesions is required, and in certain cases, surgical resection may be recommended. Of note are cystic neuroendocrine tumors, which show serous characteristics regarding cyst fluid viscosity and CEA level but are malignant.

Mucinous cystic neoplasms (MCNs) occur almost exclusively in women. On imaging, MCNs are usually characterized by unilocular cysts in the body and/or tail. Peripheral calcifications of the wall or septa can be present in about 25% of MCNs. Approximately 15% of resected MCNs contain invasive cancer, and risk factors for malignancy include size >6 cm, enhancing nodule, a thick, irregular wall, and peripheral calcifications [4,5,6]. While MCNs have malignant potential, less than 0.4% of MCNs smaller than 3 cm without a nodule harbor high-grade dysplasia or invasive cancer [7]. This suggests that not all MCNs must be resected.

IPMNs are more common with subtypes that carry different risks of malignancy. Main duct IPMNs (MD-IPMN) have the highest malignant potential (approximately 60–80%) and are characterized by diffuse or segmental dilatation of the main pancreatic duct >5 mm, forming a cystic tumor and producing mucus within the duct [8]. Branch duct IPMNs (BD-IPMNs) arise within the branches of a nondilated main pancreatic duct with a malignant risk ranging from 3% to 26% [5,8]. Mixed-type IPMNs have features of both MD-IPMN and BD-IPMN with a malignant potential comparable to MD-IPMN [9].

All main-duct and mixed-type IPMNs should be discussed for resection in patients who are fit for surgery. Absolute criteria for surgical resection of side-branch IPMNs include the presence of jaundice, contrast-enhancing mural nodule ≥5 mm, solid mass, or cytology with high-grade epithelial dysplasia or invasive carcinoma [9].

In clinical practice, the first diagnostic challenge for PCLs is to distinguish between mucinous and non-mucinous cysts. Characterization of incidental pancreatic cysts by imaging alone is limited. Gold-standard imaging to aid in the diagnosis of PCLs includes MRI pancreas with cholangiopancreatography (MRCP); however, this shows merely up to 40 to 50% accuracy in determining the cyst subtype [10]. EUS imaging has also limited diagnostic accuracy in identifying mucinous cysts, with only 51% accuracy reported [11]. However, a great advantage of EUS is its ability to obtain cystic fluid by FNA for further testing. Following EUS and before sending the fluid for biomarker analysis, the string sign should be evaluated, which is highly specific for mucinous cysts (95%) [12]. The remaining fluid should be sent for cytology, amylase, CEA, and glucose measurement. According to a recent large network meta-analysis by Li et al., FNA cytology has a sensitivity of 46% and a specificity of 89% for mucinous pancreatic cystic lesions. Intracystic CEA and glucose testing showed a sensitivity of 68% and 92%, respectively, and a specificity of 83% and 65%, respectively. Upon evaluation of KRAS (Kirsten RAt Sarcoma virus) and GNAS (Guanine Nucleotide-binding protein, Alpha Stimulating protein activity) mutational testing, the meta-analysis revealed a high specificity (93% and 99% respectively) but a low sensitivity (55% and 39% respectively) in identifying mucinous cysts [13]. The clinical benefit of KRAS/GNAS mutation analysis therefore remains uncertain at present.

The KRAS gene is an oncogene with mutations associated with several malignancies, including bronchial adenocarcinoma, colonic adenocarcinoma, and ductal adenocarcinoma of the pancreas. The first application of KRAS in the diagnosis of pancreatic cystic lesions as an intracystic genetic marker was described by Khalid A. et al. in 2005 [14]. Mutations in KRAS are known to be a major driver in pancreatic ductal adenocarcinoma progression, and targeted anti-KRAS therapies seem to be promising [15]. The GNAS gene encodes the α-subunit of the stimulatory G-protein (Gαs). Somatic activating GNAS mutation results in an elevated level of cyclic adenosine monophosphate (cAMP) and in uncontrolled growth signaling. GNAS mutation has been found in various tumors, fibrous dysplasia, and in McCune-Albright syndrome [16,17]. GNAS testing of pancreatic fluid has been shown to be a highly specific method in identifying IPMNs and MCNs [18].

In 2012, the International Association of Pancreatology (IAP) guideline designated molecular analysis as investigational and recommended only for the evaluation of small branch duct IPMNs without “worrisome features” in centers with expertise [19]. The European evidence-based guidelines on pancreatic cystic neoplasms suggest consideration of KRAS and GNAS mutation analysis in cases in which the diagnosis is unclear, and a change in diagnosis will alter management (Evidence Grade 2C, strong agreement) [9]. The guideline of the American Gastroenterological Association (AGA) considers molecular techniques as an emerging area of research and evaluates the diagnostic utility of these tests as uncertain [20].

The aim of this study was to assess the additional role of KRAS and GNAS mutation analysis with Sanger Sequencing compared to conventional cyst fluid analysis with string sign, cytology, intracystic CEA, and glucose level measurement.

## 2. Materials and Methods

This prospective study was conducted from April 2022 to July 2025; cyst fluid samples from 47 patients were collected. The study approval was obtained from the Ethics Committee of the Semmelweis University of Budapest (Ethical approval number: 1121-1/2020/EKU). All patients gave informed consent prior to the procedure for EUS with FNA to acquire intracystic fluid by EUS-FNA and PCF analysis. The clinical records, EUS, CT/MRI reports and images, cytological evaluation, cyst fluid biomarker levels, results of KRAS/GNAS mutational testing, the pathology, and surgical reports were prospectively collected and documented. The methods regarding the evaluation of morphology, FNA sampling, and cyst fluid analysis were similar to our previous study on intracystic CEA versus glucose in differentiating pancreatic cysts [21]. The diagnosis of cysts was established retrospectively by three experienced gastroenterologists (G.G., I.H., and M.H.), blinded to KRAS and GNAS results, based on cyst morphology on cross-sectional imaging and EUS, string sign, cytological characteristics, intracystic amylase, CEA, and glucose levels in non-resected cases. Applying this complex clinical assessment as a gold standard for diagnosis, the result of KRAS and GNAS mutation analysis was evaluated in identifying mucinous cysts compared to conventional testing.

### 2.1. Inclusion and Exclusion Criteria

The study cohort included patients (age > 18), after consent, who underwent EUS-FNA and cyst fluid analysis where the cyst type was unclear after imaging, or the cyst fluid analysis, including KRAS/GNAS testing, was likely to alter management in terms of differentiating between mucinous and non-mucinous cysts, or detecting dysplasia or malignancy. Exclusion criteria involved the contraindications of FNA. Cysts with obvious morphological signs of malignancy were excluded.

### 2.2. Cross-Sectional Imaging

Multidetector computed tomography (MDCT) and/or MRCP were performed for each patient. The same morphological criteria were used for cross-sectional imaging to define the assumed cyst type as for EUS.

### 2.3. EUS

The minimum cyst size recorded was 15 mm. The EUS scans were performed by two expert endoscopists (I.H. and M.H.) with a UCT-180 linear echoendoscope and an EU-ME2 Premium endoscopic ultrasound processor (Olympus GmbH) used to assess the EUS-morphology of the lesions. During EUS, documented findings included cyst location, size, number of cysts, lobularity, cyst wall thickness, septa, nodules, and solid masses associated with the cyst, pancreatic duct diameter, communication of the cyst with the pancreatic ductal system, and lymphadenopathy. The cysts were punctured with a 19/22 G EchoTip Ultra FNA needle (Cook Co., Boston, MA, USA), 2 Cysts with a Micro-Tech Areus 22 G, and 1 cyst with a Micro-Tech Trident 22 G needle (Micro-Tech Co., Nanjing, China). A 10 mL syringe with vacuum suction was used to collect the PCF. After the evaluation of the string sign, the remainder of the collected cyst fluid sample was sent for cytological analysis alongside measurement of intracystic amylase, CEA, and glucose levels. Samples from cysts with clear morphological characteristics of an IPMN on MRCP or EUS were analyzed only for string sign and cytology in some cases. No complications were observed in relation to the EUS-FNA.

### 2.4. String Sign

The string sign test was performed by placing a sample of intracystic fluid between the index finger and thumb and measuring the distance while slowly separating the fingers before the string broke. The string sign was considered positive when the string reached a minimum length of 5 mm and lasted for at least one second.

### 2.5. Cytology

For the cytological analysis, air-dried smears were prepared onto glass slides. After hematoxylin-eosin and Papanicolaou staining, the slides were assessed for the presence of malignant cells/cells with atypia, mucin- and glycogen-containing cells, inflammatory cells, and extracellular mucin.

### 2.6. CEA and Glucose

At least 1 mL of PCF was analyzed for CEA and glucose. The CEA level was measured by electro-chemiluminescence using an enzyme-labelled sandwich immunoassay; the glucose level was measured by spectrophotometric assessment using Hexokinase. To differentiate between mucinous and non-mucinous PCLs, we applied the traditionally used cut-off value of 192 ng/mL for CEA, and the standardized cut-off value of 2.8 mmol/L (50 mg/dL) for glucose levels [11,22].

### 2.7. Amylase

We considered an intracystic amylase level under 250 U/L as an exclusion criterion for pseudocysts.

### 2.8. KRAS and GNAS Mutational Analysis

A minimum of 0.5 mL fluid was used for mutational tests. The samples were immediately transported for genetic testing, and, in our study, to examine the GNAS and KRAS mutation status, a HighPur PCR Template Kit (Roche) was used to isolate DNA and intronic sequence-specific primers were used to amplify the entire length of exon 2 of KRAS (NM_004985.5) and exon 8 of GNAS (ENST00000371085.8). The purified PCR fragments of the PCR products were analyzed by direct sequencing in both sense and antisense directions. The sequencing reaction was performed with the BigDye^®^ Terminator v1.1 Cycle Sequencing Kit (Applied Biosystems™, Foster City, CA, USA—by Life Technologies™, Carlsbad, CA, USA) according to the manufacturer’s protocol (same primers as the ones used for PCR amplification reactions). Before analysis, purification of the sequencing reaction products was performed by the BigDye^®^ XTerminatorTM Purification Kit (Applied Biosystems ™—by Life Technologies™). PCR products were analyzed by a SeqStudio Genetic Analyzer (Thermo Fisher Scientific, Waltham, MA, USA). In the initial phase of the study, we performed KRAS exon 2 mutation analysis (19 patients) by Sanger sequencing and subsequently performed both KRAS and GNAS exon 8 testing (28 patients). The molecular analysis was assessed by a molecular pathologist blinded to clinical, imaging, cytological, and intracystic glucose and CEA results.

### 2.9. Follow-Up

The study was conducted from April 2022 to July 2025; the median follow-up time was 252 (751−80) days in median.

### 2.10. Statistical Analysis

Data collection, evaluation, and figure generation were performed using GraphPad Prism (GraphPad Software Inc., Boston, MA, USA, version 10.6.1) and Microsoft Office Excel (Microsoft Corporation, Redmond, WA, USA, version Microsoft 365) software. Normality tests (Anderson–Darling, D’Agostino and Pearson, Shapiro–Wilk, Kolmogorov–Smirnov) were performed to determine the normality level of the samples. In instances where a normal distribution was observed, an independent two-sample *t*-test (Student) was employed for intergroup comparisons. For continuous variables that deviated from a normal distribution, non-parametric tests (Mann–Whitney) were utilized for comparative investigations. Categorical variables were characterized by specifying the number of elements in the corresponding category and calculating the percentage distribution. For statistical analyses, we utilized the χ2 test or, in cases of low (less than 5) expected values, Fisher’s exact test. Continuous variables were presented as mean ± standard deviation (SD) or median with range (IQR), as appropriate for the data distribution. All statistical tests were conducted assuming a two-tailed distribution, with a significance threshold set at *p* < 0.05.

## 3. Results

The total sample size consisted of 47 patients, with a distribution of 15 men and 32 women. A broad spectrum of pancreatic cystic lesions was identified, with 33 mucinous lesions: 24 IPMNs, 5 mucinous cystadenomas (MCAs), and 4 adenocarcinomas. Fourteen non-mucinous lesions were included: five pseudocysts/walled off necroses (WONs), eight serous cystadenomas (SCAs), and one cystic neuroendocrine tumor (cNET). Twenty-six cysts were unilocular and twenty-one were multilocular. The median diameter of the largest cyst was 35.5 mm (48.5−25.0). The baseline patient and cyst characteristics are displayed in Table 1.

**Figure 1:**
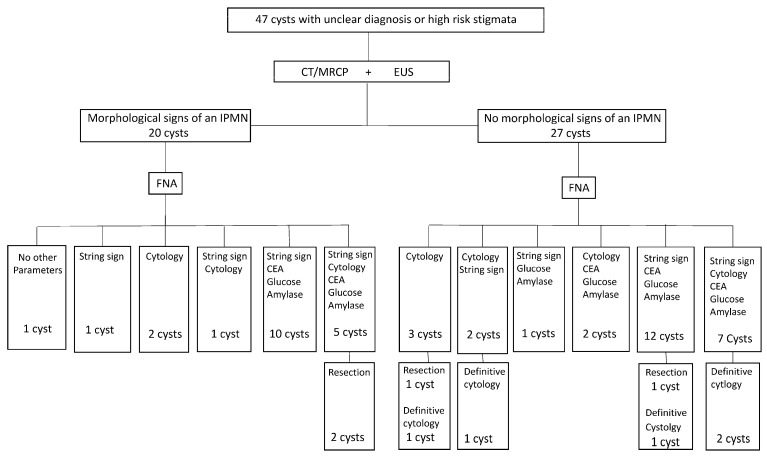
Diagnostic strategy.

Four patients (8.5%) underwent surgical resection with corresponding histology reported as one pancreatic intraepithelial neoplasia (panIN) with high-grade dysplasia, one IPMN with low-grade dysplasia, one IPMN with high-grade dysplasia, and one MCN. Cytological analysis of PCF additionally revealed a definite diagnosis in five cases (10.6%): four adenocarcinomas and one cNET. These lesions were not resected because of unresectability or refusal of the patient. The remaining 38 cases (80.9%) were diagnosed based on imaging, cytological characteristics, intracystic CEA, and glucose level. The diagnostic strategy is displayed in Figure 1.

The string sign could be unequivocally assessed in 38 cases (82.9%). Cystic fluid for cytology was obtained in 45 patients (95.7%). The quality of the investigated samples was satisfactory in 22 (48.8%), suboptimal in 17 (37.7%), and unsatisfactory in 6 (13.3%) cases. A CEA result was recorded in 36 (76.6%) and glucose level in 37 (78.7%) patients. CEA and glucose were intentionally not measured in the case of eight cysts because of suggestive morphology for IPMN or adenocarcinoma. The CEA measurement was not successful in three cases. The diagnostic and intracystic marker characteristics are displayed in Table 2.

Genetic testing for KRAS mutations was performed in all 47 cases. GNAS testing was additionally performed in 28 of these cases. KRAS mutations were detected in 14 out of the 33 mucinous cysts (sensitivity of 42.4%, 0.2724 to 0.5919, CI 0.95). GNAS mutations were detected in 10 out of 23 mucinous cysts (sensitivity of 43.4%, 0.2563 to 0.6319, CI 0.95). The specificity for diagnosing mucinous cysts was found to be 92.9% (0,6853 to 0,9963 CI 0.95) for KRAS and 100% (0.5655 to 1.000 CI 0.95) for GNAS. In the six mucinous lesions with histologically or cytologically proven high-grade dysplasia or malignancy, KRAS mutations were identified in five cases (83.3%). GNAS was tested in five patients in this subgroup, in which mutations could be detected in two cases (40%). The single diagnosed cNET showed no evidence of KRAS or GNAS mutations. The results are summarized in Table 3 and Table 4. 

The application of molecular analysis altered the clinical management significantly in one patient. In this case, conventional testing supported the diagnosis of a serous cyst; however, a KRAS mutation was detected, and therefore, close follow-up has been planned with the potential for repeat FNA, dependent on eventual morphological changes. Additionally, in two other patients, where the measurement of the traditionally used biomarker, CEA, was not successful, positive mutational analysis results supported the diagnosis of a mucinous cyst. However, in these cases, other parameters such as the morphology, the string sign, and the cytology were also suggestive of a mucinous etiology.

## 4. Discussion

Differentiation of pancreatic cysts continues to be a diagnostic challenge, which will likely remain in the near future. The expanded use of cross-sectional imaging has resulted in increased detection of asymptomatic pancreatic cysts; however, the ability of these imaging methods to accurately define the cyst subtype is limited. Certain PCLs, such as MCNs and IPMNs, have the potential for malignant transformation. Whilst underestimation of the potential malignant risk of PCLs may lead to undetected carcinoma development, overestimation can cause a significant financial burden on healthcare systems, unnecessary follow-up examinations, or superfluous morbidity and mortality through unnecessary surgical procedures. Key steps in the work-up of PCLs include, primarily, the differentiation between mucinous and non-mucinous lesions, and secondly, the differentiation between low-risk and high-risk lesions. To help determine the subsequent management, the combination of morphological characteristics on EUS and cross-sectional imaging, the string sign, cytology, intracystic CEA, and glucose measurement is conventionally applied as a standard in daily clinical practice.

Molecular analysis of pancreatic cystic fluid has been reported to be possibly useful in identifying mucinous cysts and detecting neoplasia, with a recent systematic review and meta-analysis by McCarty et al., including six studies (785 lesions) reporting a sensitivity of 75% and specificity of 99% in identifying IPMNs and mucinous cystadenomas by using combined KRAS and GNAS testing. However, this method did not prove to be sufficient for diagnosing high-risk or malignant PCLs [18]. In a network meta-analysis by Li et al., using definitive histological and pathological diagnoses as a gold standard, the sensitivity and specificity in identifying mucinous lesions by combined KRAS + GNAS analysis were 77% and 93% respectively, and 73% and 55%, respectively, for malignant cysts [13]. Other biomarkers, such as methylated DNA markers, mucins, SMAD4, and RNF43 proved to be more effective for detecting malignancy [23].

Some former studies with relatively small sample sizes expressly assessed the added clinical value of KRAS and GNAS testing. Faias et al. analyzed 52 frozen samples of pancreatic cyst fluid obtained by EUS-FNA. In addition to cytology and CEA, mutations of KRAS (exons 2 and 3) and GNAS (exons 8 and 9) were evaluated using Sanger sequencing. After molecular testing, a correct modification in cyst classification occurred merely in two patients, and the molecular analysis was assessed to have no significant diagnostic benefit in comparison with conventional testing [24]. Herranz Pérez et al. obtained samples from 36 pancreatic cysts and analyzed them for cytology, CEA, glucose, and various genetic markers, including KRAS and GNAS. Only six lesions were classified as non-mucinous; however, two of them showed mutations in KRAS and GNAS, recategorizing these lesions as mucinous neoplasms, which led to a modification of the follow-up plan [25].

The aim of our present study was to assess the clinical utility of KRAS and GNAS mutation analysis in identifying mucinous lesions.

Limitations of this study include that the collected data are from a single center and the sample size is relatively small. From this, only a small proportion of cases (19.1%) had definitive cytology or surgical histology results. However, we consider the combination of morphological assessment, string sign, cytology, cyst fluid CEA, and glucose as a reliable reference standard. In this regard, the cyst population in our study represents a wide spectrum of cysts with various sizes and morphologies, which may correlate better with everyday clinical practice than that of surgical cohorts. Additionally, in 17% of the cases, no cytology sample was obtained, or it was unsatisfactory for analysis. Furthermore, CEA and glucose level measurements were not performed or were not successful in 23.4% and 21.3% of the cases, respectively. Finally, in the initial phase of the trial, we performed KRAS testing alone, combining KRAS and GNAS testing only later in the study. Our results showed a significantly lower sensitivity for KRAS compared to some previously published data [13,18]. This may be explained by the limitations of Sanger sequencing compared to Next-Generation-Sequencing (NGS) in pancreatic cyst evaluation.

Supposing a high specificity of the mutational analysis in identifying mucinous cysts, we changed the clinical management in one patient. This cyst showed non-mucinous characteristics on conventional testing; however, a KRAS mutation was detected. Considering a mucinous etiology, a close follow-up has been planned with potential for repeating the cyst fluid sampling. In two other patients, the intracystic CEA measurement was not successful; however, the mutational analysis supported the diagnosis of mucinous cysts. This beneficial characteristic of the mutational analysis—requiring low fluid volume and not being influenced by high cyst fluid viscosity—may be advantageous in clinical practice, because these factors may impede the CEA measurement.

## 5. Conclusions

We found the sensitivity of mutational analysis for KRAS and GNAS inferior to conventional testing in detecting mucinous cysts; however, the application of the genetic testing may alter the clinical management. Due to their high specificity, the detection of mutations in these alleles may also be useful in supporting the diagnosis of a mucinous cyst in uncertain cases.

## Figures and Tables

**Table 1:** Patient and cyst characteristics.

	All (*n* = 47, 100%)	Mucinous (*n* = 33, 70.2%)	Non-Mucinous (*n* = 14, 29.8%)	*p*-Value
**Patient demographics**				
				
Gender				
Male	15 (31.9%)	13 (39.4%)	2 (14.3%)	0.17
Female	32 (68.1%)	20 (60.6%)	12 (85.7%)	
Age in years, median (IQR)	65.8 (±14.8)	68.7(±14.7)	58.9 (±13.1)	0.04
**Cyst characteristics**				
Localization of the largest cyst, *n*, (%)				0.85
Uncinate process	1 (2.1%)	1 (3.0%)	0 (0.0%)	
Head	20 (42.6%)	13 (39.4%)	7 (50%)	
Genu of pancreas	7 (14.9)	4 (12.1%)	3 (21.4%)	
Body	14 (29.8%)	11 (33.3%)	3 (21.4%)	
Body–tail border	2 (4.3%)	2 (6.1%)	0 (0.0%)	
Tail	3 (6.4%)	2 (6.1%)	1 (7.1%)	
				
Largest cyst diameter in mm, median (IQR)	35.5 (48.5−5.0)	32.0 (41.0−25.0)	47.5 (67.0−24.0)	0.11
Locularity				0.46
Unilocular cyst, *n* (%)	26 (55.3%)	18 (54.5%)	8 (57.1%)	
Multilocular cyst, *n* (%)	21 (44.7%)	15 (45.5%)	6 (42.9%)	

**Table 2:** Diagnostic and intracystic marker characteristics.

Diagnosis	Mucinous	Non-Mucinous	All	*p* Value
SCN	8 (17.0%)	0 (0.0%)	8 (57.1%)	<0.0001
MCN	5 (10.6%)	5 (15.2%)	0 (0.0%)	
IPMN	24 (51.1%)	24 (72.7%)	0 (0.0%)	
Adenocarcinoma	4 (8.5%)	4 (12.1%)	0 (0.0%)	
WON/Pseudocyst	5 (10.6%)	0 (0.0%)	5 (35.7%)	
NET	1 (2.1%)	0 (0.0%)	1 (7.1%)	
Biomarker characteristics				
CEA (>192 ng/mL), median (IQR)	46.1 (215.5−1.6)	55.5 (338.4−23.9)	0.7 (113.0−0.5)	0.02
Amylase (250 IU/L), median (IQR)	210 (32,360.0−123.5)	3078 (30,900.0−601.8)	99.0 (43,096.0−22.0)	0.06
Glucose (50 mg/dL), median (IQR)	11.7 (90.0−11.7)	11.7 (11.7−11.7)	100.8 (108.0−71.2)	<0.0001

**Table 3:** Results.

Cyst Type	All	Mucinous	Non-Mucinous
KRAS			
wild	32 (68.1%)	19 (57.6%)	13 (92.9%)
mutant	15 (31.9%)	14 (42.4%)	1 (7.1%)
GNAS			
wild	18 (38.3%)	13 (39.4%)	5 (35.7%)
mutant	10 (21.3%)	10 (30.3%)	0 (0.0%)
not analyzed	19 (40.4%)	10 (30.3%)	9 (64.3%)

**Table 4:** Results.

K-Ras	Value	(95% CI) p0.02
Sensitivity	0.4242	0.2724 to 0.5919
Specificity	0.9286	0.6853 to 0.9963
Positive Predictive Value	0.9333	0.7018 to 0.9966
Negative Predictive Value	0.4063	0.2552 to 0.5774
**GNAS**	**Value**	**(95% CI) p0.13**
Sensitivity	0.4348	0.2563 to 0.6319
Specificity	1.0	0.5655 to 1.000
Positive Predictive Value	1.0	0.7225 to 1.000
Negative Predictive Value	0.2778	0.1250 to 0.5087

## Data Availability

Data supporting reported results are not available due to privacy restrictions.

## Abbreviations

PCL	Pancreatic cystic lesion
KRAS	Kirsten Rat Sarcoma Virus
GNAS	Guanine Nucleotide-binding protein, Alpha Stimulating protein activity
PCF	Pancreatic cystic fluid
EUS	Endoscopic ultrasound
FNA	Fine needle aspiration
CEA	Carcinoembryonic antigen
SCA	Serous cystadenoma
MCA	Mucinous cystadenoma
MCN	Mucinous cystic neoplasm
IPMN	Intraductal papillary mucinous neoplasm
MD-IPMN	Main duct intraductal papillary mucinous neoplasm
BD-IPMN	Branch duct intraductal papillary mucinous neoplasm
cAMP	Cyclic Adenosine Monophosphate
AGA	American Gastroenterological Association
CT	Computed Tomography
MRI	Magnetic resonance imaging
MRCP	Magnetic resonance cholangiopancreatography
NGS	Next-generation sequencing
